# Retraction Note: Assessing survival time of outpatients with cervical cancer: at the university of Gondar referral hospital using the Bayesian approach

**DOI:** 10.1186/s12905-026-05036-3

**Published:** 2026-10-24

**Authors:** Chalachew Gashu, Buzuneh Tasfa, Chekol Alemu, Yoseph Kassa

**Affiliations:** 1 Department of Statistics, College of Natural and Computational Science, Oda Bultum University, Chiro, Ethiopia; 2 Department of Epidemiology, Faculty of Public Health, Jima University, Jima, Ethiopia; 3 Department of Statistics, College of Natural and Computational Science, Gambella University, Gambella, Ethiopia

**Retraction Note: BMC Women’s Health (2023) 23:59**

**https://doi.org/10.1186/s12905-023-02202-9**

The Editors have retracted this Article.

Following publication, concerns were raised regarding the authors’ affiliations, the omission of a supervisor who should have been included as an author, the use of ethical clearance obtained from an unauthorized body, and potential concerns regarding the authenticity and reliability of the reported data. Upon request, the Authors acknowledged the omission of their supervisor and confirmed that they did not list an affiliation with the University of Gondar, where the research was conducted. The Authors were unable to provide a satisfactory explanation regarding the data concerns and could not supply documentation of valid ethics approval.

The Editors therefore no longer have confidence in the reliability of the content of this Article.

Chekol Alemu and Buzuneh Tasfa disagree with the retraction and its wording. Chalachew Gashu agrees with this retraction but not with the wording of the retraction notice. Yoseph Kassa disagrees with this retraction.

